# Quantitative assessment of myocardial perfusion by magnetic resonance imaging in the isolated porcine heart

**DOI:** 10.1186/1532-429X-13-S1-P55

**Published:** 2011-02-02

**Authors:** Andreas Schuster, Amedeo Chiribiri, Masaki Ishida, Matthias Paul, Shazia Hussain, Niloufar Zarinabad Nooralipour, Geraint Morton, Roy Jogiya, Divaka Perera, Tobias Schaeffter, Sven Plein, Eike Nagel

**Affiliations:** 1 King's College London BHF Centre of Excellence, Division of Imaging Sciences, NIHR Biomedical Research Centre at Guy's and St. Thomas' NHS Trust Foundation, Wellcome Trust EPSRC Medical Engineering Centre; The Rayne Institute, St. Thomas´ Hospital, London, UK; 2 King's College London BHF Centre of Excellence, Division of Imaging Sciences, The Rayne Institute, St. Thomas´ Hospital & University of Leeds, Multidisciplinary Cardiovascular Research Centre, G-floor Jubilee Wing, Leeds General Infirmary, Leeds LS1 3EX, London & Leeds, UK

## Objective

To validate quantitative myocardial perfusion CMR against known myocardial blood flow rates in the isolated perfused porcine heart.

## Background

Perfusion imaging visualizes early changes within the ischaemic cascade and has been shown to accurately detect significant coronary artery stenoses. First pass myocardial perfusion CMR yields high spatial resolution and permits quantification of myocardial blood flow. However, perfusion-CMR has only been validated in a small number of previous studies and using relatively early perfusion methods. An isolated blood-perfused MR compatible porcine heart model which can be imaged by identical equipment used for humans has been recently proposed. The model permits full control of regional myocardial blood flow, thus providing a valuable tool for the validation of quantitative perfusion assessment.

## Methods

9 perfusion experiments were performed in male white cross landrace pigs (54±6 kg). Hearts were harvested under terminal anaesthesia and transported to the laboratory under cold cardioplegic arrest. After reperfusion the coronary flow rate was set by an external pump to accommodate stable sinus rhythm. Myocardial blood flow of 0.8 ml/min/g was considered normal reference flow of 100%.

Hearts were positioned in a clinical 1.5 Tesla MR Scanner (Philips Achieva) and myocardial perfusion CMR was performed with a k-t SENSE accelerated high-resolution balanced Turbo Field Echo pulse sequence yielding a spatial resolution of 1.7x1.9x10 mm. Perfusion-CMR was performed using a dual bolus scheme (5 ml of neat (7mmol/l) and 5 ml of dilute (0.7 mmol/l) gadobutrolum, Gadovist, Schering, Berlin, Germany) and quantified using Fermi function constrained deconvolution. Values were expressed as Mean±SD per slice (6 region of interests) and compared with the known coronary flow rate.

## Results

**Figure 1:**
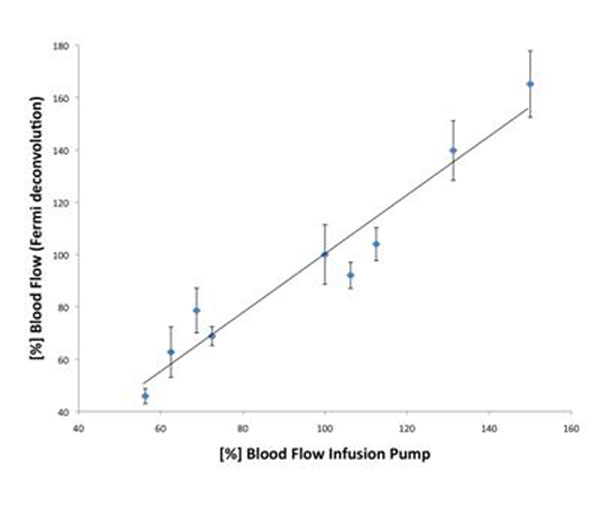
Relation between [%] Myocardial Blood Flow given by the infusion pump and [%] Blood Flow derived from magnetic resonance perfusion imaging (Fermi deconvolution).

Hearts remained stable during isolated coronary perfusion and thus experiments were successfully conducted. Mean myocardial blood flow of the external pump was set to 96±33%. There was good correlation with mean myocardial perfusion as assessed by CMR (95±38%) using simple linear regression analysis (r=0.95, mean difference 0.35%, limits of agreement +24.43% and -23.74%). Figure 1 shows the quantitative result of each experiment in regard to the externally set blood flow.

## Conclusion

Quantitative assessment of myocardial perfusion using CMR correlates well with known myocardial blood flow in an isolated porcine heart model. Further studies using fluorescently labelled microspheres as the gold-standard are required to confirm this finding.

